# Effects of Sound Frequency on Audiovisual Integration: An Event-Related Potential Study

**DOI:** 10.1371/journal.pone.0138296

**Published:** 2015-09-18

**Authors:** Weiping Yang, Jingjing Yang, Yulin Gao, Xiaoyu Tang, Yanna Ren, Satoshi Takahashi, Jinglong Wu

**Affiliations:** 1 Department of Psychology, Faculty of Education, Hubei University, Hubei, China; 2 Biomedical Engineering Laboratory, Graduate School of Natural Science and Technology, Okayama University, Okayama, Japan; 3 Bio-robotics and System Laboratory, Beijing Institute of Technology, Beijing, China; 4 School of Computer Science and Technology, Changchun University of Science and Technology, Changchun, China; 5 Department of Psychology, School of Philosophy and Sociology, Jilin University, Changchun, China; University of Rome, ITALY

## Abstract

A combination of signals across modalities can facilitate sensory perception. The audiovisual facilitative effect strongly depends on the features of the stimulus. Here, we investigated how sound frequency, which is one of basic features of an auditory signal, modulates audiovisual integration. In this study, the task of the participant was to respond to a visual target stimulus by pressing a key while ignoring auditory stimuli, comprising of tones of different frequencies (0.5, 1, 2.5 and 5 kHz). A significant facilitation of reaction times was obtained following audiovisual stimulation, irrespective of whether the task-irrelevant sounds were low or high frequency. Using event-related potential (ERP), audiovisual integration was found over the occipital area for 0.5 kHz auditory stimuli from 190–210 ms, for 1 kHz stimuli from 170–200 ms, for 2.5 kHz stimuli from 140–200 ms, 5 kHz stimuli from 100–200 ms. These findings suggest that a higher frequency sound signal paired with visual stimuli might be early processed or integrated despite the auditory stimuli being task-irrelevant information. Furthermore, audiovisual integration in late latency (300–340 ms) ERPs with fronto-central topography was found for auditory stimuli of lower frequencies (0.5, 1 and 2.5 kHz). Our results confirmed that audiovisual integration is affected by the frequency of an auditory stimulus. Taken together, the neurophysiological results provide unique insight into how the brain processes a multisensory visual signal and auditory stimuli of different frequencies.

## Introduction

In everyday life, our brain receives many sensory signals, such as vision or sound. The integration of information from different sensory modalities is an essential component for cognition. Previous studies have shown that responses to bimodal audiovisual stimuli are faster and more accurate compared with unimodal auditory or visual stimuli presented alone [1–3]. This beneficial effect between visual and auditory stimuli is generally referred to as “audiovisual integration” [4, 5].

Indeed, the audiovisual facilitative effect strongly depends on stimulus features, such as the spatial frequency or the contrast of visual stimuli [6–8]. Sound has two basic acoustic features: intensity and frequency. For intensity (decibel, dB), behavioral studies have demonstrated that lower sound intensities lead to more audiovisual facilitation than higher sound intensities [9, 10]. Moreover, event-related potential (ERP) studies have shown that audiovisual integration is elicited by the lower intensity stimuli but not the higher intensity stimuli in 40–60 ms after stimulus presentation when visual and auditory stimuli are simultaneously attended [7]. With regards to sound frequency, it is the most ubiquitous feature to which cortical neurons are tuned in the auditory system. Some studies have shown that the primary auditory cortex could select sound information based on sound frequency-content [11], suggesting that sound frequency plays a significant role in auditory processing. Some researchers have put forth hypotheses about the encoding process for sound frequency [12, 13]. There are currently two theories about pitch encoding: the place theory (encoding based on the site of activation along the cochlea) and the temporal theory (encoding based on the phase-locked activity of hair cells and auditory neurons). Moreover, characteristic frequency topographical mapping has found pitch-selective neurons near the anterolateral border of the primary auditory cortex in animals [14] and in humans [15, 16]. Furthermore, the neural mechanisms underlying the processing of changes in sound frequency have also been studied in the human brain [17, 18]. For example, an ERP study found that the latency of the P300 component became shorter as the difference between the standard (1 kHz) and target tone frequency increased (1.5, 2, 4 kHz) [19]. To date, however, relatively little is known about the interactions between auditory stimuli of different frequencies and visual stimuli.

Behavioral studies have shown that a higher frequency sound embedded in a sequence of lower frequency sound improved the detection of synchronously presented visual targets [20]. However, whether a higher frequency sound presented alone can enhance the detection of visual stimuli remains unclear. Furthermore, it was also not clear how sounds of different frequencies modulate integration between visual and auditory stimuli from electrophysiological evidence. To investigate this question, we designed a visual target detection task that included auditory stimuli of different frequencies. Here, we studied the nature and timing of audiovisual integration occurring with different sound frequencies using the high temporal resolution of EEG. We found out fundamental patterns of influence of sound frequency, one of the basic characteristics of auditory stimuli, on audiovisual integration.

## Materials and Methods

### Participants

Fourteen healthy volunteers (aged 21–27 years, mean age 24.2 years) were recruited as paid volunteers. All participants had normal or corrected to normal vision and were right-handed. The participants provided written informed consent for their participation in this study, which was previously approved by the ethics committee of Okayama University.

### Stimuli and task

Stimulus presentation and response collection were accomplished using Presentation software (Neurobehavioral Systems Inc., Albany, California, USA). The experiment consisted of three stimulus types: unimodal visual, unimodal auditory and bimodal audiovisual (auditory and visual stimuli that occurred simultaneously).

As described in detail previously [5], the unimodal visual stimulus was a Gabor patch with gratings and included two subtypes: standard and target stimuli. The unimodal auditory stimuli consisted of 65 dB sound- pressure level (SPL), which was measured using an SPL meter (Galaxy Corporation, California, USA). The auditory stimuli were presented for 40 ms (10 ms rise and fall times) through an earphone (CX-300, Sennheiser, Japan). The sound frequencies included 0.5, 1, 2.5 and 5 kHz. The selection of these frequencies was motivated by previous studies [21, 22]. Moreover, some previous investigations of audiovisual integration adopted lower frequencies [1, 5, 23], but based on the relationship between SPL and frequency presented in Robinson D (1957), a relatively higher frequency (5 kHz) was selected in our study. Bimodal audiovisual stimuli were presented at four levels: visual stimulus (standard or target) with auditory stimuli of 0.5 kHz (A_0.5_V), 1 kHz (A_1_V), 2.5 kHz (A_2.5_V) and 5 kHz (A_5_V). The target stimuli were presented at a frequency of approximately 12.5% of the total stimuli.

**Fig 1:**
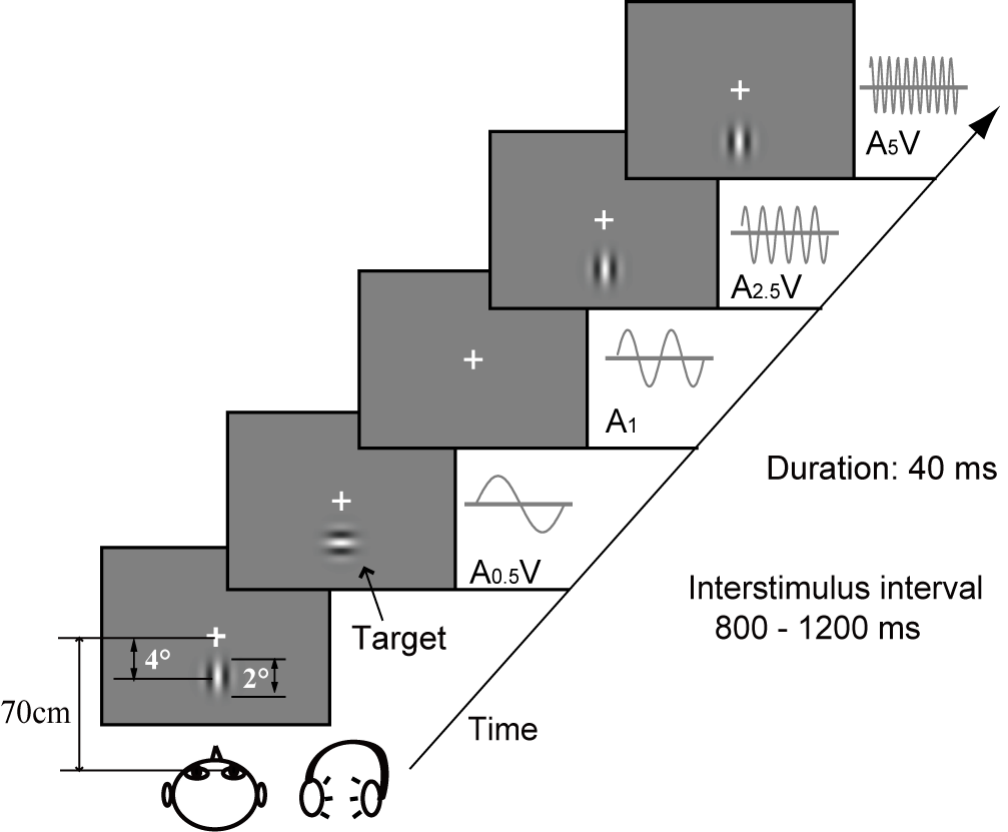
Experimental design. Stimuli were presented in a random and continuous stream of unimodal visual stimuli, unimodal auditory stimuli at four different frequencies and audiovisual stimuli. The auditory stimuli of 65 dB were presented through earphones. The visual target stimulus was a Gabor patch with horizontal gratings. Participants sat approximately 70 cm from the screen, and the subject’s task was to make a quick and accurate button response when the visual target stimulus was presented, regardless of whether an auditory stimulus was presented. A_0.5_, A_1_, A_2.5_ and A_5_: frequency of auditory stimuli of 0.5, 1, 2.5 and 5 kHz, respectively.

In this study, there were 350 (280 + 70) unimodal visual stimuli, 1120 (280 × 4) unimodal auditory stimuli, and (280 × 4 + 70 × 4) audiovisual stimuli. All stimuli were presented with a randomly varying inter-stimulus interval (ISI; measured from the offset of one trial to the onset of the next) of between 800 and 1200 ms (mean = 1000 ms). During the experiment, as shown in Fig 1, the participants fixated a cross on a screen. The participants’ task was to respond to visual target stimuli as quickly and accurately as possible using their right hand, regardless of whether an auditory stimulus was presented.

### Apparatus

Electroencephalographic (EEG) signals were recorded at a sampling rate of 500 Hz from 30 scalp electrodes (Easy-cap, Herrsching Breitbrunn, Germany). The electrodes were referred to the left and right earlobes. Horizontal and vertical eye movements were also recorded by deriving the electrooculogram (EOG). The impedance of all electrodes was below 5 kΩ. Brain Vision Analyzer software (version 1.05, Brain Products GmbH, Munich, Bavaria, Germany) was used to analyze the ERPs, which were averaged separately for each stimulus type off-line.

### Data Analysis

#### Behavioral Data

The response time was measured based on the timing of the participant’ button presses in response to the presented stimuli. The hit rate was defined as the number of correct responses to target stimuli divided by the total number of target stimuli. The false alarm rate was calculated as the number of responses to the standard stimuli divided by the total number of standard stimuli. In addition, signal detection analysis was applied to compute sensitivity measures (d’) and criterion (c) separately for each stimulus type to disentangle the effects of detection sensitivity and response bias [24, 25]. In the formulas below, *H* corresponds to the hit rate, *F* to the false alarm rate and Φ^-1^ to the inverse of the normal cumulative distribution function:

$$d'=\Phi^{-1}(H)-\Phi^{-1}(F);\;c=-(\Phi^{-1}(H)+\Phi^{-1}(F))/2$$

Differences in response times, hit rates, false alarm rates, d’ and c of the participants were analyzed using a repeated measures analysis of variance (ANOVA). The level of significance was fixed at a corrected *p* < 0.05.

#### ERP data Analysis

See reference [5] for detail about the fundamental analysis of EEG signals. The difference wave [AV-(A+V)] was quantified as the audiovisual integration effect [26, 27]. In other words, audiovisual integration was the difference between the ERPs to bimodal (AV) stimuli and the ERPs to the sum of the unimodal stimuli (A+V). Previous studies have also investigated audiovisual integration using this method [28–31].

**Fig 2:**
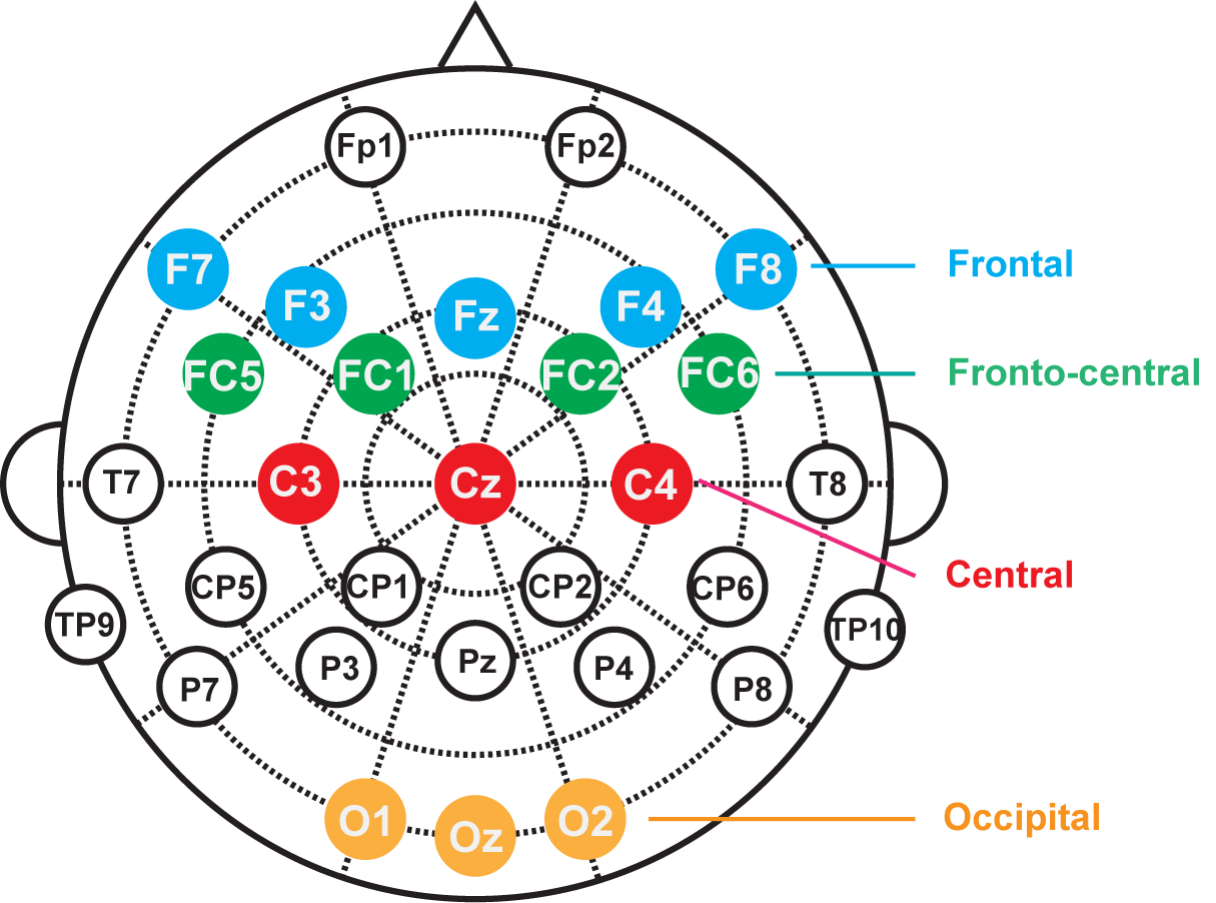
EEG electrode placement and the 4 ROIs.

To establish the presence of audiovisual interaction, we conducted three phases of analysis for the ERPs. The first phase of analysis was performed to render a full description of the spatio-temporal properties of the audiovisual integration. Thus, point-wise running t-tests (two-tailed) were used to compare AV with (A+V) for each scalp electrode under each sound frequency condition. In the present data treatment, periods of significant difference were only plotted if an alpha criterion was less than 0.05 and then only if this criterion was exceeded for at least 12 consecutive data points (12 data point = 24 ms at a 500 Hz digitization rate) (see, [1, 32–34]). Then, four regions of interest (ROI) (frontal: F7, F3, Fz, F4, F8; fronto-central: FC5, FC1, FC2, FC6, central: C3, Cz, C4 and occipital: O1, Oz, O2) were selected based on statistical analysis and the topographical response pattern (Fig 2). In the second phase of analysis, repeated measures ANOVAs were conducted separately for the four frequencies of auditory stimuli for the time intervals that were selected based on an overview of the significant differences in the first phase of analysis. The mean amplitude data were analyzed with within-subjects factors of stimulus type (AV, A+V) and ROI (frontal, fronto-central, central and occipital). If a significant interaction between stimulus type and ROI was observed for the main time intervals, the third phase of analysis would be performed. In the third phase of analysis, the ANOVAs were measured separately for each of the four ROIs using the factor stimulus type (AV, A+V). The SPSS version 16.0 software package (SPSS, Tokyo, Japan) was used for all statistical analyses.

## Results

### Behavioral Results

Table 1 shows the reaction times. Repeated-measures ANOVA of the mean response times revealed a significant difference between the unimodal visual and the bimodal audiovisual stimuli [F(4, 52) = 10.508, *p* < 0.001]. Post-hoc t-tests revealed that responses to bimodal audiovisual stimuli, which included 0.5 kHz (*p* < 0.01), 1 kHz (*p* < 0.01), 2.5 kHz (*p* < 0.01) and 5 kHz (*p* < 0.001) stimuli, were faster than those to the unimodal visual stimuli, but there was not a significant difference between the sound frequencies (*p* = 1.0). However, no significant effects for hit rates [F(4, 52) = 1.799, *p* = 0.158] and false alarm rates [F(4, 52) = 1.583, *p* = 0.215] were found for unimodal visual and bimodal audiovisual stimuli (Table 1). Perceptual sensitivity (d’) and response bias (c) are also shown in Table 1. There was no significant effect of stimulus type on d’ [F(4, 52) = 1.179, *p* = 0.330] or c [F(4, 52) = 1.730, *p* = 0.176].

**Table 1:** Behavioral mean data over all participants in the experiment.

Stimulus types	Reaction time (ms)	Hit rate (%)	False alarm rate (%)	Perceptual sensitivity (d’)	Response criterion (c)
V	521 (14.6)	82.6 (3.7)	0.36 (0.11)	3.66 (0.15)	0.81 (0.08)
A_0.5_V	506 (13.3)	85.6 (3.1)	0.61 (0.18)	3.67 (0.16)	0.70 (0.07)
A_1_V	505 (12.5)	84.0 (3.7)	0.43 (0.13)	3.69 (0.13)	0.75 (0.08)
A_2.5_V	501 (14.5)	85.8 (2.9)	0.38 (0.19)	3.83 (0.14)	0.78 (0.07)
A_5_V	502 (13.0)	84.1 (2.8)	0.33 (0.11)	3.71 (0.11)	0.81 (0.07)

Standard error of the mean (SEM) is given in parentheses. A_0.5_V, A_1_V, A_2.5_V, A_5_V: auditory stimulus of bimodal audiovisual stimuli was 0.5, 1, 2.5, 5 kHz, respectively; V: unimodal visual stimuli.

### ERP Results

#### Event-related potential: unimodal stimuli

**Fig 3:**
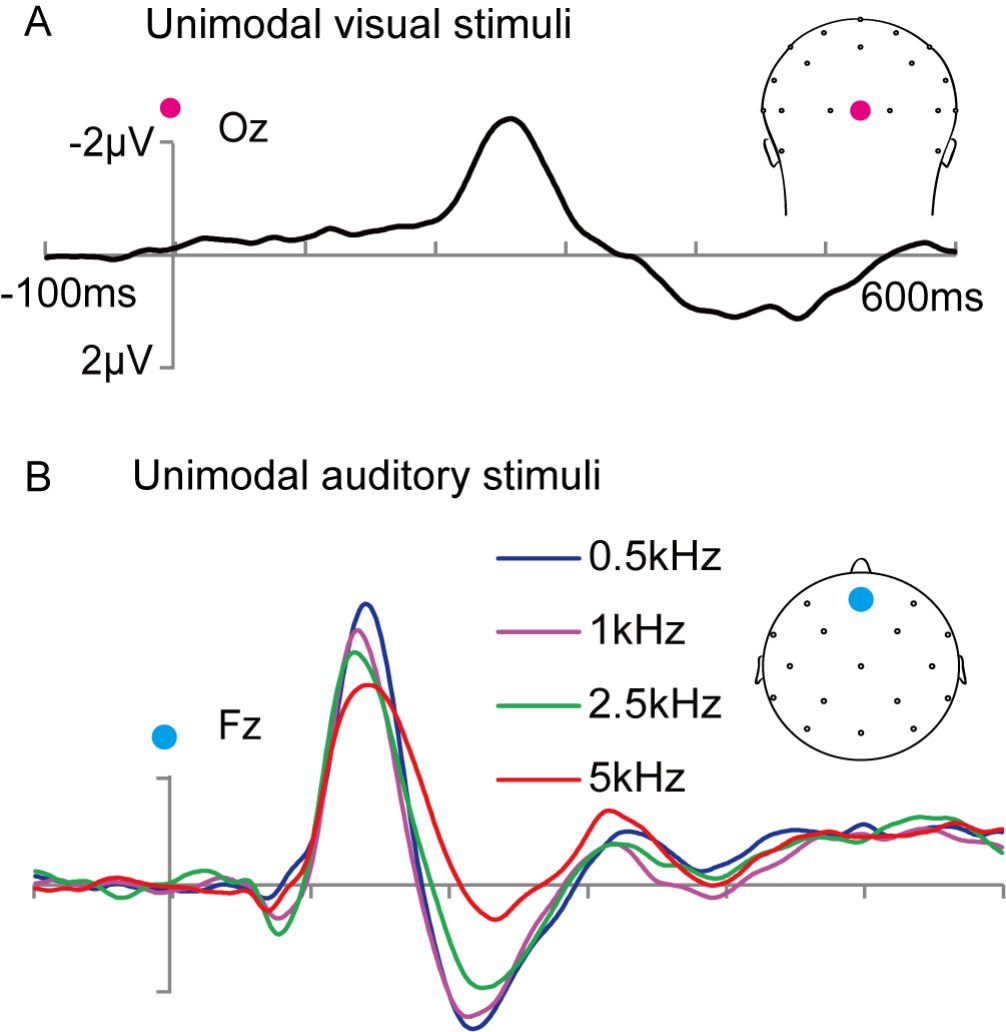
Waveforms of unimodal stimuli. Unimodal visual (A) and auditory stimuli (B). 0.5, 1, 2.5, 5: frequency of unimodal auditory stimuli is 0.5, 1, 2.5, 5 kHz, respectively.

The group-averaged ERPs to the unimodal visual stimuli and the unimodal auditory stimuli are shown in Fig 3. The peak of the visual ERP was -2.41 μV at Oz, and the ERP contained a prominent negative wave that peaked approximately 260 ms after stimulus onset at the occipital sites (Fig 3A). For the four different frequencies of auditory stimuli (0.5, 1, 2.5 and 5 kHz), the ERPs showed a negativity-polarity wave peaking at approximately 140 ms (-5.27 μV), 134 ms (-4.77 μV), 130 ms (-4.34 μV) and 140 ms (-3.74 μV) at Fz, respectively (Fig 3B). Apparently, the amplitude of the negative component decreased with increasing sound frequency.

#### Point-wise running t-tests for electrodes

**Fig 4:**
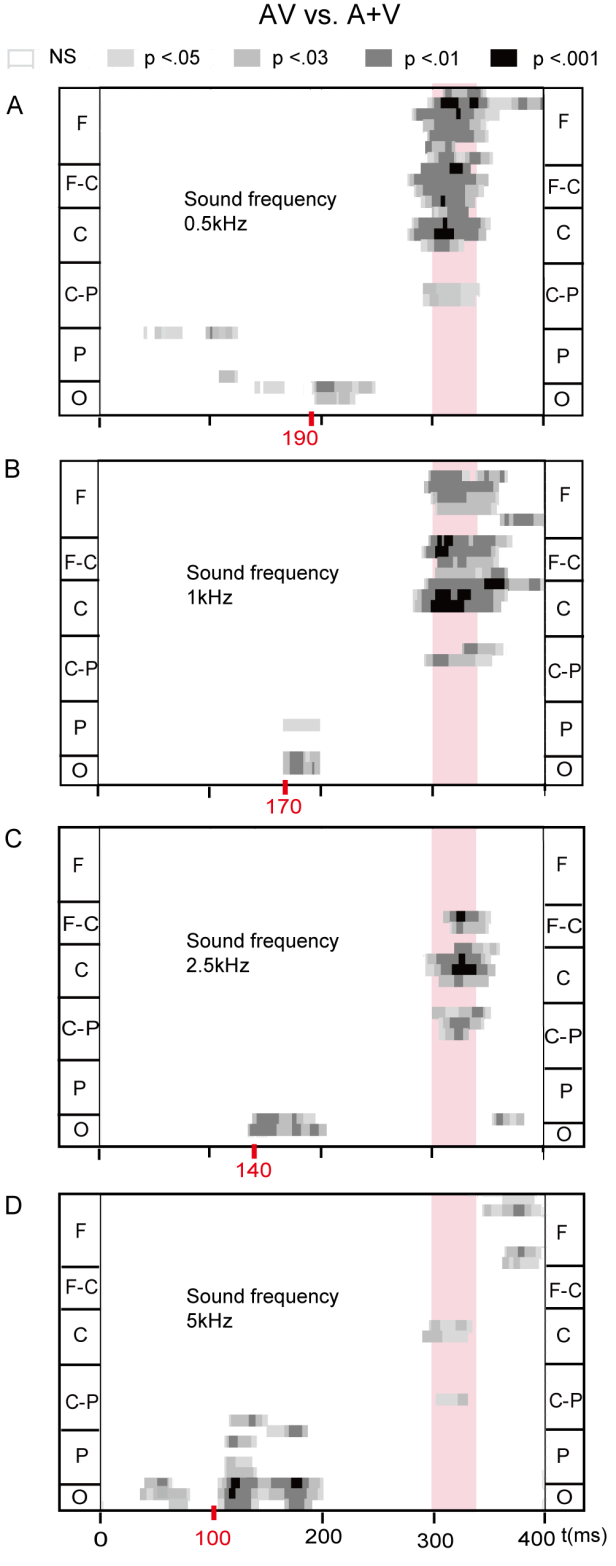
Statistical significance of audiovisual integration. The effects from point-wise running t-tests comparing AV to (A+V) for all participants when sound frequency is 0.5 kHz (A), 1 kHz (B), 2.5 kHz (C), 5 kHz (D), respectively. Time is plotted on the x-axis from 0 ms to 400 ms. Electrodes are plotted on the y-axis. Within a section the electrodes are arranged from the left lateral to the right lateral sites. Red points are the earliest start time of integration. Pink shades: integration effects at the late stage. F, frontal; F-C, fronto-central; C, central; C-P, centro-parietal; P, parietal; O, occipital.

Fig 4 shows the results of the point wise running t-tests (AV vs A+V) when auditory stimuli were presented at four different frequencies. The time intervals that were selected for the region of interest (ROI) analysis are highlighted with red numbers and pink shading. For early integration, the onset time of audiovisual interaction was different in the occipital area when auditory stimuli were presented in the four conditions; 190 ms for 0.5 kHz, 170 ms for 1 kHz, 140 ms for 2.5 kHz, 100 ms for 5 kHz. It was clearly observed that integration effects occurred earlier with increasing sound frequency. For approximately 300–340 ms, an obvious pattern of integration effects was visible at fronto-central and central electrode sites for sound frequencies of 0.5, 1 and 2.5 kHz. Audiovisual integration effects were analyzed in detail in the ROI analysis described below.

#### Audiovisual Integration at occipital area (100–210 ms)

**Fig 5:**
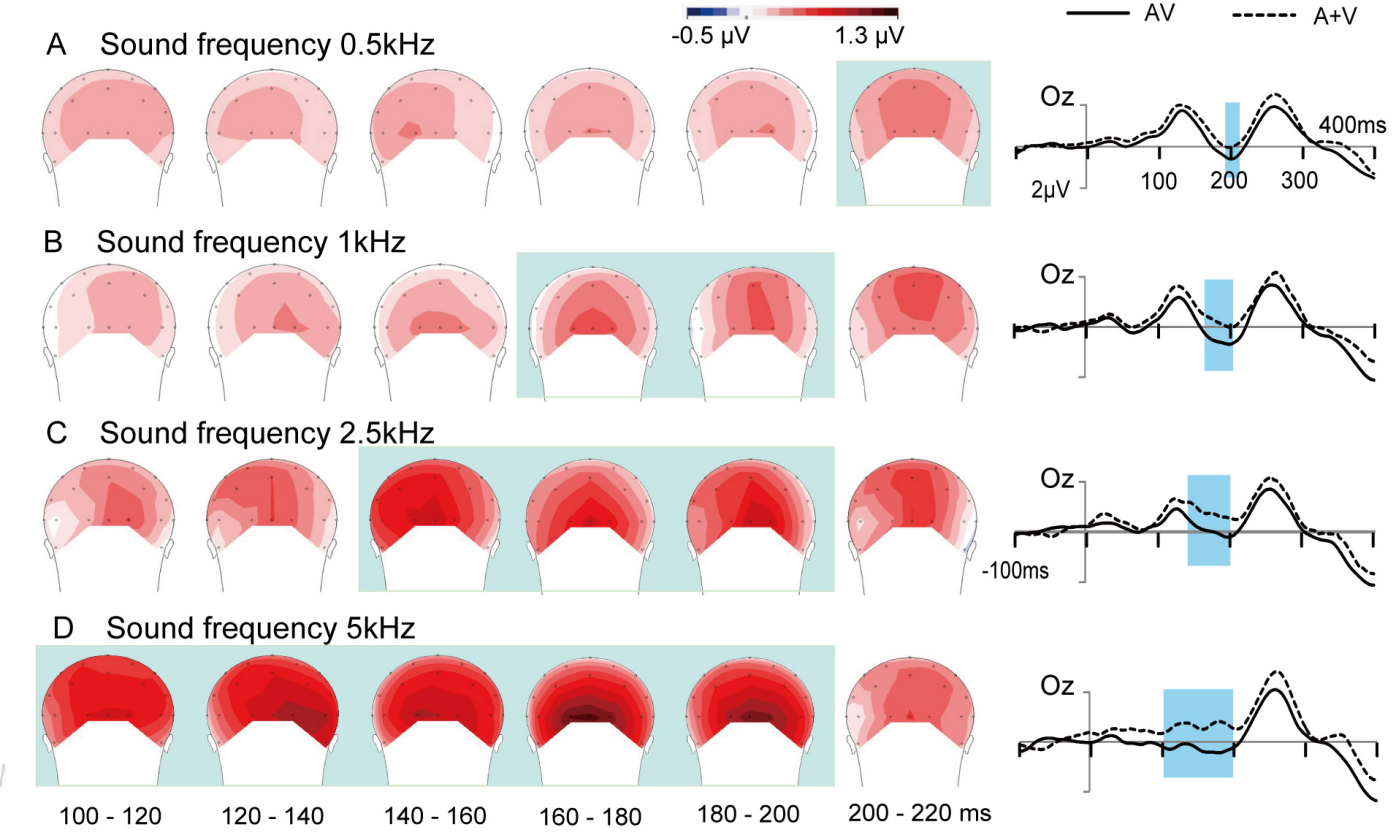
Topography of the significant spatio-temporal patterns of integration in the occipital area. The time of onset of audiovisual integration was different when auditory stimuli were presented in the four conditions (A) 0.5 kHz, (B) 1 kHz, (C) 2.5 kHz, (D) 5 kHz. Right sides: Event-related potential of the sum of the unimodal stimuli (A+V) and bimodal (AV) stimuli at a subset of electrodes are shown from 100 ms before the stimulus to 400 ms after. The shade areas indicate the time periods when the bimodal response significantly differs from the sum of the unimodal responses (*p* < 0.05).

For early audiovisual integration, the observed integration effects at the time of onset were notably different in the four conditions at the occipital electrodes (O1, Oz and O2) (Fig 4). Thus, ANOVAs were performed separately for the four sound frequency conditions using the factor stimulus type. The results showed that occipital electrodes had a significant main effect of stimulus type (AV and A+V) for auditory stimuli of 0.5 kHz in 190–210 ms [F(1, 13) = 7.720, *p* = 0.016], 1 kHz in 170–200 ms [F(1, 13) = 11.845, *p* = 0.004], 2.5 kHz in 140–200 ms [F(1, 13) = 11.574, *p* = 0.005], and 100–200 ms [F(1, 13) = 14.450, *p* = 0.002]. Moreover, the topographies also show this integration effect in Fig 5. Furthermore, Fig 5 (right side) shows the ERPs to AV and (A+V) waveforms at Oz in four conditions. This finding is of particular interest because it shows that the effects of higher frequency auditory stimuli on audiovisual integration processes can occur earlier in time.

#### Audiovisual integration for 300 to 340 ms

**Fig 6:**
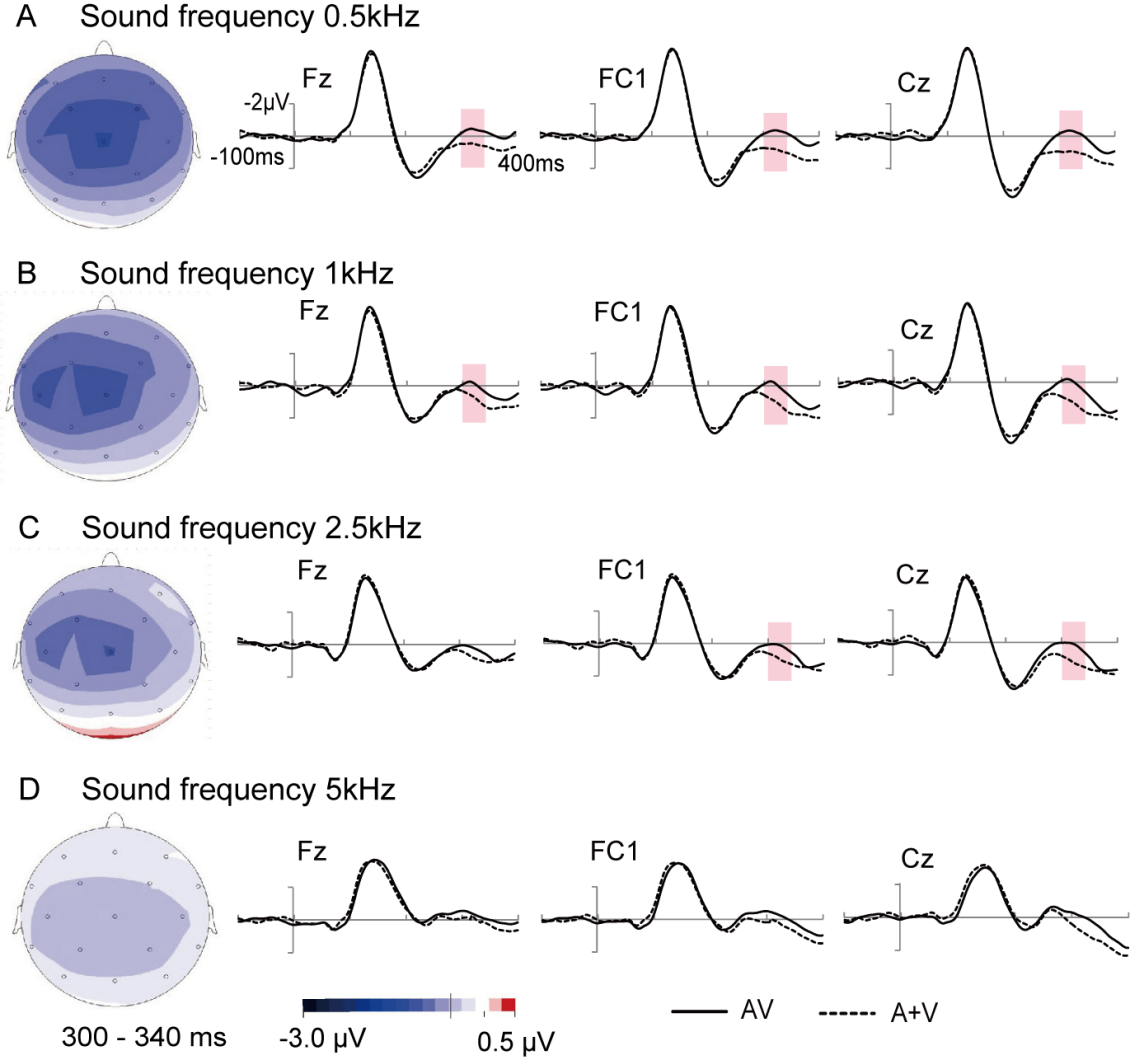
Topography of the different significant spatio-temporal patterns of integration in the frontal and central areas. An obvious pattern of integration effects was visible at 300–340 ms for sound frequencies of (A) 0.5 kHz, (B) 1 kHz and (C) 2.5 kHz at fronto-central and central areas; (D) No similar pattern of integration effects was observed for 5 kHz sound frequency at approximately 300–340 ms.

For auditory stimuli of 0.5 kHz, an ANOVA using the factors stimulus type (AV and A+V) and ROI (frontal, fronto-central, central and occipital) revealed a significant interaction between the two factors [F(3, 39) = 13.914, *p* = 0.001]. Follow-up ANOVAs were measured separately for the different ROIs using the factor stimulus type. These ANOVAs showed significant main effects of stimulus type for 0.5 kHz at the frontal [F(1, 13) = 20.628, *p* = 0.001], fronto-central [F(1, 13) = 18.642, *p* = 0.001] and central areas [F(1, 13) = 16.099, *p* = 0.001]. The amplitudes of the frontal, fronto-central, and central sites were more negative in AV (mean potential: -0.07 μV, -0.16 μV, -0.21 μV, respectively) compared with (A+V) (mean amplitude: 0.89 μV, 0.97 μV, 1.02 μV, respectively) (Fig 6A). Furthermore, the topographies of [AV-(A+V)] also showed differences at the frontal, fronto-central, and central areas due to smaller amplitudes in AV than in (A+V) (Fig 6A).

When the frequency of the auditory stimulus was 1 kHz, a significant interaction was found between stimulus type and ROI [F(3, 39) = 10.913, *p* = 0.002]. ANOVAs were implemented separately for ROIs. Significant main effects of stimulus type were revealed at the frontal [F(1, 13) = 10.321, *p* = 0.007], fronto-central [F(1, 13) = 14.584, *p* = 0.002] and central area [F(1, 13) = 14.137, *p* = 0.002]. As shown in Fig 6B, the amplitudes were smaller in AV than (A+V) [AV—(A+V): -0.8 μV, -0.99 μV and -1.08 μV]. Furthermore, topographies of brain activity are also shown in Fig 6B.

The integration effect was analyzed using similar ANOVAs on ROI for 2.5 kHz auditory stimuli. These analyses yielded a significant interaction between stimulus type and ROI at 300–340 ms [F(3, 39) = 13.035, *p* = 0.001]. Then, ANOVAs were measured separately for the ROIs using the factor stimulus type. Analysis of these amplitudes showed a main effect of stimulus type at the fronto-central [F(1, 13) = 8.184, *p* = 0.013] and central area [F(1, 13) = 18.383, *p* = 0.001]. Fig 6B illustrates this effect, in which the amplitudes at both fronto-central and central electrodes were smaller in AV (0.27 μV, 0.18 μV) than (A+V) (1.04 μV, 1.19 μV). In addition, the topographies of the integration effect appeared at fronto-central and central areas (Fig 6C). In contrast to lower frequency sound, the ANOVAs for 5 kHz sound did not reveal any significant effects for 300–340 ms post-stimulus onset (Fig 6D).

## Discussion

Our study clearly showed that sound frequency affects audiovisual integrative processing in evoked brain activity. For audiovisual integration at the early stage (100–210 ms), it was observed that integration effects over the occipital area occurred earlier when the sound frequency was higher. For approximately 300–340 ms, an obvious pattern of integration effects was visible at the fronto-central area for lower sound frequencies (0.5, 1 and 2.5 kHz); however, for higher frequency sound (5 kHz), a similar pattern of integration effects was absent at 300–340 ms post-stimulus.

### Integration at the early stage

The novel finding of this study is that integration effects occur earlier with increasing sound frequency, as early as 100 ms after stimulus onset (Figs 4 and 5). This phenomenon may be related to the pitch encoding of pure tones. Although the neural code for the pitch of pure tones is still a matter of debate, two theories about pitch encoding have been postulated [35, 36]. The first is the place code theory, which is based on the site of maximum excitation of the cochlea. The cochlea is a spiral-shaped tube filled with fluid, making 2.5 turns around its axis in humans [37]. The higher sound frequencies are processed at the base, and the lower sound frequencies are processed at the apex, which means that sensory cells are arranged successively from high to low frequencies along the entire length of the cochlea [38, 39]. The second is the temporal theory, which is based on the periodicity in the temporal firing patterns of auditory neurons, or phase-locking. The initial temporal pitch code in the auditory periphery is converted to a code that is based on neural firing rate in the brainstem [40]. Other researchers have suggested that place and temporal information may be combined to form a spatio-temporal code for pitch [41–43]. In addition, previous studies have found that the time lags between auditory and visual stimuli affect integration [44, 45]. Temporal importance was also confirmed in several studies about multisensory integration, which found that the respond time tends to be shorter when auditory stimuli were presented in close temporal and spatial proximity [46, 47]. These findings suggest that visual and auditory signals may be integrated or processed faster if the visual and auditory stimuli occurred closer in time. Thus, the process of pitch encoding may be one of the possible reasons for our observations, wherein visual stimuli that were presented with higher frequency sound elicited earlier audiovisual integration, and integration gradually occurred later as sound frequency decreased.

Another possible reason might be related to the loudness of the stimulus. Higher frequency sounds (5 kHz) tend to be perceived as louder than lower frequency sounds, without normalization [48, 49]. Some auditory neuroimaging studies found that the perceived loudness of auditory stimuli increased with increasing BOLD signal strength in the auditory cortex; higher BOLD signal strength is correlated with faster processing of auditory stimuli [50, 51], resulting in earlier audiovisual integration. However, in the present study, only present frequencies ranging from 0.5 to 5 kHz; thus, our study does not allow us to draw conclusions about how high a sound frequency is needed to evoke an early integration effect. Further electrophysiological studies are needed to elucidate the neural mechanisms of integration under more detailed sound frequency conditions.

In the present study, integrations at the early stage occurred in the occipital area (Fig 5). Some functional magnetic resonance imaging (fMRI) and EEG studies have shown that audiovisual integration can occur not only in established multisensory integration regions of the brain but also in regions that were traditionally considered sensory specific (e.g., visual cortex) [52–54]. Moreover, direct anatomical connections between the superior temporal (auditory processing) and occipital regions (visual processing) in animals [55] and humans [56] have been confirmed that may play an important role in audiovisual integration. Thus, in the current study, audiovisual integration was elicited in the occipital area, which is in agreement with the findings obtained from a previous study [28].

### Integration at the late stage (300 to 340 ms)

Major integration was found in the 300–340 ms interval in the fronto-central area for lower frequency sound (0.5, 1 and 2.5 kHz) (Figs 4 and 6). Previous ERP studies investigated audiovisual integration in visual attention while ignoring auditory stimuli of 1.6 kHz [57, 58]. In line with our results, the authors reported that audiovisual integrations occurred over fronto-central scalp regions. In addition, in auditory ERP studies, fronto-central scalp topography is related to auditory attention. Attended auditory stimuli elicited an enhanced positivity component over fronto-central sites [59]. Furthermore, a source localization technique confirmed the neural generators of brain activity elicited by auditory attention were existed in the auditory cortex [60]. Accordingly, the frontal/fronto-central distribution of audiovisual integration might be due to attention to the auditory signal. However, in the current study, the participants were asked to ignore the auditory stimuli. What could cause this phenomenon? In particular, Busse et al (2005) investigated the effect of attention to visual stimuli on task-irrelevant auditory stimuli based on a visual discrimination task. The results showed that unattended sounds with visual stimuli could also cause brain activity, which was larger when simultaneously presented visual stimuli were attended versus unattended. Their ERP results revealed that the attention-related difference was over the frontal and fronto-central scalp regions. Moreover, fMRI results also confirmed the specific facilitation effect of activity in the auditory cortex, indicating that this effect was produced by an unattended sound signal [61]. Therefore, these findings indicated that attention to visual stimuli spreads to auditory stimuli, resulting in enhanced activity in the auditory cortex [61]. Donohue et al (2011) further confirmed that attention, which spread from the visual to the auditory modality when the stimuli were simultaneous, affects brain activity at fronto-central areas and that this effect occurs in a relatively later stage (200–700 ms) [62]. These findings suggest that brain activity elicited by an auditory signal at a late latency might be due to the auditory signal getting attention from attended visual stimuli. Our results are analogous to findings obtained from previous studies, in which the facilitation effect occurred relatively later over fronto-central areas.

By comparing the characteristic of sound, we found that the lower frequency sound used in this study was similar in frequency to the sound used in previous studies. In our study, the unattended lower frequency sound signals were 0.5, 1 and 2.5 kHz, respectively, whereas a tone of 1.2 kHz was used in previous studies [61, 62]. Therefore, audiovisual integration at the late stage might have occurred due to unattended auditory stimuli of lower frequencies obtaining attention from attended visual stimuli, owing to spreading attention required timing. However, when higher sound frequencies were presented, the human brain might have processed the signals faster. Thus, it is possible that audiovisual integration was completed early when the auditory stimulus was 5 kHz. In other words, the audiovisual integration that occurs during the early and late stages may overlap, and leading to more efficient effect even though the facilitative effects were smaller for 5 kHz.

## Conclusions

We showed that sound frequency can modulate audiovisual integration. We found that integration effects occurred earlier when sound frequency was higher. The earliest integration effects began at 100 ms after stimulus onset in the occipital area when task-irrelevant auditory stimuli were 5 kHz, suggesting that a higher frequency sound signal paired with a visual stimulus might be processed or integrated early. Furthermore, integration effects at longer latencies were observed in widespread scalp regions involving frontal, fronto-central and central areas in the lower frequency auditory stimuli conditions, indicating that the attended visual signal spread attention to unattended lower frequency auditory stimuli, apparently reflecting late enhanced processing of auditory information. Our results provide compelling evidence for audiovisual integration between visual and auditory channels in different sound frequency conditions. We believe that our findings are likely to be a useful reference for further studies that investigate how the integration mechanism is affected by stimulus features.

## Supporting Information

S1 Fig The waveform of AV and (A+V) from -100 ms to 700 ms in the frontal-central areas for 5 kHz. (TIF)

S1 File The hearing test results of all the participants. (XLS)

S2 File Raw data of 1–7 participants. (ZIP)

S3 File Raw data of 8–14 participants. (ZIP)
