# Supplementary figures and images for: Effects of Sound Frequency on Audiovisual Integration: An Event-Related Potential Study
Source: PLoS One. 2015 Sep 18;10(9):e0138296. doi: 10.1371/journal.pone.0138296 (PMC4575110; doi:10.1371/journal.pone.0138296)

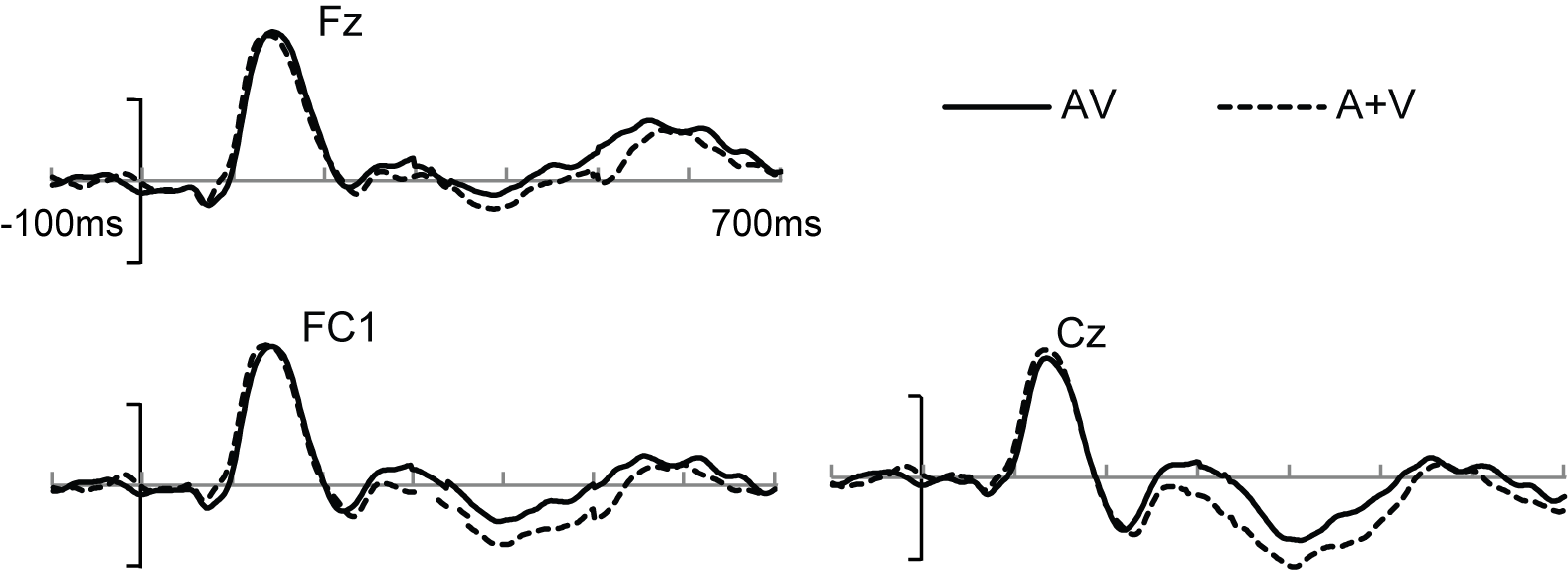

Supplement: S1 Fig — (TIF) [file pone.0138296.s001.tif]
